# Participation Motives of Sport and Exercise Maintainers: Influences of Age and Gender

**DOI:** 10.3390/ijerph17217830

**Published:** 2020-10-26

**Authors:** Clemens Ley

**Affiliations:** 1 Institute for Sport Science, University of Vienna, 1150 Vienna, Austria; clemens.ley@univie.ac.at; 2 Department of Health Sciences, University of Applied Science FH Campus Wien, 1100 Vienna, Austria

**Keywords:** physical activity promotion, motivation, behaviour change, sport participation, exercise adherence, self-determination theory

## Abstract

Promotion of physical activity has become a global priority for public health. While many people do not adhere to the recommendations, sport and exercise maintainers have found their *right* or *fitting* practice. Thus, assessing and knowing the participation motives across maintainers helps to improve our understanding of the sports and exercise practices and, thus, to recommend and to design physical activities and programmes that fit to individuals’ motives. A modified version of the Bernese Motive and Goal Inventory was used in an Austria-wide cross-sectional study with 10646 sport and exercise maintainers (43% female). The study performed confirmatory factor analysis, examined measurement invariance, and compared participation motives. The results showed a good model fit and measurement invariance, indicating that the inventory can be applied independently of gender, age and years of sport/exercise experiences. Motives differed among gender, age and type of sports/exercise. Therefore, these variables should be considered in tailoring sport recommendations and interventions to promote adherence. Finally, the results are discussed by using the Self-Determination Theory indicating that sport and exercise maintainers pursue predominantly motives with intrinsic goal contents.

## 1. Introduction

Promotion of physical activity behaviour, including regular sport and exercise practice, has become a global priority for health promotion and prevention [1]. There is now relatively good evidence of the health benefits of sport and exercise [2] and the negative effects of physical inactivity [3]. Nonetheless, international and regional recommendations on physical activity behaviour are poorly adhered to worldwide [1] and the burden of inactivity is high [3]. Thus, it is crucial to improve our understanding on how to promote adoption and maintenance of physical activity [4]. Bauman et al. [5] question in their review of physical activity correlates: why some people are physically active and others not? The current study focuses on those who are physically active in order to learn from their successful maintenance of sport and exercise behaviour. Participation motives have been shown to be one important factor to understand physical activity behaviour [6,7,8]. Recommending the type of sport/exercise fitting to the individual motive profile may promote maintenance of physical activity; cf. [9,10]. Furthermore, gender and age correlate with physical activity behaviour [5,11], intention [12] and motivation [13,14,15,16]. Women tend to be less active than men throughout the lifespan and engage in less vigorous-intensity activities [5,11,17], which relates to different health benefits [17]. Gender and age are expected to influence participation motives in sport and exercise maintainers as well. Thus, the current study examines gender and age invariance of a measurement instrument and differences regarding participation motives in sport and exercise maintainers.

### 1.1. Theoretical Concepts and Considerations

While inactive people fail to do sport and exercise due to multifaceted individual, social and environmental factors [4,5,15,18], sport and exercise maintainers excel through regular sport and exercise practices over an extended period of time and through a strong motivation and volition for the maintained practice of their respective sport or exercise [19,20]. Maintainers have most probably found their *right* or *fitting* sport or exercise practice, i.e., the subjective incentives of the chosen activity fit or fulfil the individual’s motives for participation; cf. [9]. The subjective incentives are of a prompting nature, inciting for the reach of the expected goals or outcomes [9,10]. Thus, knowing the participation motives of maintainers of different sports and exercise practices helps us to improve our understanding of the subjective incentives in the respective sport and exercise practice; cf. [21]. In a first step, this knowledge allows a more comprehensive description of the activities, adding a subjective category to the currently predominantly objective categorizations (e.g., individual/team sports, outdoor/indoor, aerobic/anaerobic exercises, high/low risk). A comprehensive description is required for recommending the *right* and *fitting* sport/exercise. In a second step, this knowledge allows the development of strategies for keeping people physically active; for example, by designing and tailoring sport and exercise programmes that satisfy to a higher degree the subjective needs and motives of their participants [22,23]. This may contribute to reduce dropout rates and fluctuation in sport participation and promote maintained physical activity behaviour [18,22,23]. 

The participation motives refer to the subjective goal contents of sport and exercise participation, as a result these two terms, *motives* and *goal contents*, are often used interchangeable [18,24]. People have multifaceted motives for participating in sport and exercise, which range, for example, from health management to skills development and to social recognition. 

Studies reported differences according to gender and age [25,26]. Women give more importance to psychological benefits [6,27], physical condition [26] and physical appearance [6,26,27]. Zervou et al. [27] showed that potential improvements in physical appearance were central for female adults to participate in exercise programmes. However, this may not be the case for overweight participants, ”mainly due to the notion that their body image is considered significantly different to the prevailing social norms” [27] (p. 8), indicating the moderating role of social acceptance for participation and of the perceived *ideal* body image. Conversely, Cash et al. [28] indicated that appearance/weight management motives relate to negative body image independent of body mass; and Ednie and Stibor [29] showed that appearance (but not weight management) motive was a negative predictor for exercise participation in females. Men give more importance to competition/ego [25,26,27,30], mastery [26] and others’ expectations [6,26]. Age correlates positively with the importance of health-related motives and inversely with the importance of competition-related motives [25,26,27,30] and affiliation [26]. Trujillo et al. [31] differentiated four age groups and conclude that ”health may be an importance incentive for exercisers of all ages, while appearance may be an important incentive for only young and early middle-age individuals” (p. 362). Less is known about differences according to sport and exercise types [22,26,32]. Practitioners of team sports seem to rank more important the social and affiliation motives and individual sports practitioners the competition, performance and challenge related motives, whereas exercisers value health-, fitness- and appearance-related motives as more important [22,26,33]. Hence, age, gender and type of sport and exercise are important factors that influence individuals’ choice and physical activity behaviour. Thus, differentiated knowledge on the motive structures of these different groups is useful for tailoring recommendations and programmes [26]. This is particularly important as the fulfilment of motives is considered one important step towards a maintained sport and exercise practice [8,34], as displayed in the Self-Determination Theory (SDT) [10,18].

SDT is a well-established theory that helps us to understand and explain motivation and physical activity behaviour [18]. SDT distinguishes between *goal contents* or (*participation*) *motives* and the *regulatory processes* underlying the pursuits of the goal contents, that is, between the *what* (goal contents) and the *why* (regulatory process) of goal pursuits [24]. The regulatory processes derive from the basic psychological needs (*autonomy*, *competence* and *relatedness*) and direct people’s goal pursuits, e.g., through more intrinsic and internalised regulations or through more external regulations [24]. Furthermore, the motives can also be differentiated according to more *intrinsic* or more *extrinsic* goal contents. *Intrinsic* goal contents (e.g., health management, skill development, challenge, social affiliation) strongly relate to the fulfilment of basic psychological needs and are inherently satisfying to pursue. *Extrinsic* goal contents rather relate to substitute needs and show an outward orientation (e.g., appearance/image, social recognition) [18,24,35]. Ingledew et al. [10] showed that the different participation motives predicted different regulation processes: appearance/weight predicted external and introjected regulation; health- and stress-related motives predicted identified regulation; affiliation and challenge motives predicted intrinsic regulation; and social recognition predicted external regulation in young adults; cf. [29]. According to SDT, sport and exercise maintainers would pursue predominantly intrinsic goal contents as these relate more to the fulfilment of the basic psychological needs and to sustained behaviour [7,8,22,26]. Furthermore, motives can also be classified according to the expected reward, i.e., if the action of sport participation in itself is rewarding (autotelic, task and action-related goal contents; e.g., experiencing a flow state or positive affects, mastering a challenge) or if the outcome of sport participation is rewarding (instrumental, outcome-related goal contents; e.g., connecting to new people). 

### 1.2. Assessment of Participation Motives

Several questionnaires assess motives and goal contents. They differ in the range of motives covered and in their alignment to SDT. For example, the widely used *Exercise Motive Inventory* (EMI-2) covers 51 items of a particular broad range of participation motives [36]. The *Goal Content for Exercise Questionnaire* (GCEQ) is limited to 20 items that align more closely to the SDT by focusing more strictly on the *what* (goal contents) of the goal pursuits [37]. The *Bernese Motive and Goal Inventory* (BMZI) [38] aims to capture the full range of motives of people in middle adulthood (35–65 years) in health and recreational sport and exercise. The BMZI includes 24 items based on seven dimensions, i.e., *Fitness/Health, Body/Appearance, Contact (in and through sports), Distraction/Catharsis, Activation/Enjoyment, Competition/Performance* and *Aesthetics*. It is considered suitable for an economic individual assessment and useful to design individual- or group-tailored sport programmes and recommendations [23,38]. In a recently updated version of the BMZI [23], which was published after completion of the present study, the BMZI was adapted by deleting the *Activation/Enjoyment* dimension and differentiating *Health* and *Fitness*. According to the authors, this revised version can be connected with SDT as it focuses more strictly on the goal contents [23]. Ley and Krenn [30] applied the BMZI in a sample of sports performers of a broader range of age (18–65 years) as in the original BMZI. The findings of confirmatory factor analysis and cross validation suggested a slight modification of the BMZI for this target group. Two items (‘for the thrill’ and ‘primarily out of joy of movement’) were excluded; consequently, the coverage of the factor *Activation/Enjoyment* was questioned content-wise [30]. In total, the various versions of the BMZI showed acceptable to good model fits [23,30,38]. Furthermore, Schmid et al. [23] showed measurement invariance of the updated BMZI across less and more active people. Measurement equivalence regarding gender, age and years of sport and exercise experiences, and the sensitivity to depict group differences according to these variables is widely unknown for sport and exercise maintainers. Scalar measurement invariance would suggest that the measurement model is suited for comparison of the participation motives among gender, age and years of sport/exercise experiences and, thus, is a prerequisite for the further analysis and application of the questionnaire.

### 1.3. Purpose of the Study and Hypothesis

The purpose of the study is to contribute (a) to the provision of a validated questionnaire that allows measuring motive differences among age, gender and years of sport/exercise experiences, and (b) to the understanding of the goal contents of (diverse groups of) sport and exercise maintainers and, thus, of the subjective incentives of the respective sport and exercise practice. In total, the study aims to contribute to the discussion of strategies for keeping people physically active, considering individual and group characteristics, particularly gender and age, for tailoring interventions and recommendations to fit to the individual motives. Therefore, the study analyses (a) the factorial validity and measurement invariance of the BMZI in sport and exercise maintainers, and (b) the participation motives across groups of maintainers. The study has the following hypotheses: Firstly, acceptable model fits of the inventory and invariance across gender, age and years of sport/exercise experiences are expected [23,30,38]. Secondly, participation motive means differ among maintainers: It is expected that men give more importance to competition-related motives than women [25,26,27,30]; that the importance of health-related motives increases and the importance of competition-related motives decreases with age [25,26,27,30]; and that maintainers of team sports are more contact-oriented, and individual exercisers more fitness/health-oriented [22,26,33]. Thirdly, it is expected that maintainers pursue predominantly goal contents related to intrinsic motivation [22,26].

## 2. Materials and Methods 

### 2.1. Design and Instrument

This cross-sectional study was conducted in Austria from 2013 to 2017, targeting adult sport and exercise maintainers. In this study, *maintainers* were defined as people who sustain the practising of a sport/exercise regularly at least one hour per week and for at least one year. In comparison to other authors and models like the Trans-Theoretical Model, which used a six-month period for defining maintenance [19,20,26], this study took a more conservative approach, also due to seasonal variations in sport participation. 

Data on a broad range of sports and exercise practices were collected through the online survey platform *LimeSurvey*. Forty sports and exercise practices were included in this study, ranging from team sports (e.g., football, basketball, ultimate Frisbee) to individual sports/exercises with a direct opponent (e.g., racquet sports, martial arts) and with no direct opponent (e.g., running, exercising in a gym, surfing), but excluded sports with minimal physical activity (e.g., darts or pool). For each of the 40 sports/exercise practices, a separated online survey was established. The questionnaire included the motives for participating in the respective sport, using the BMZI with its 24 items and a five-point Likert scale. The original BMZI [38] and an English translation are available in Schmid et al. [23]. Questions regarding the participation motives always referred to the specific sport/exercise (‘I’m doing X because/to...’) and goal contents (‘to be social with other’; ‘because of my body shape’, ‘to compete with others’). 

Furthermore, the questionnaire included the questions asking for age, gender and self-reported sport and exercise participation specific data (i.e., frequency, duration and years of practising the sport, subjective skills level) as independent variables. The diverse sports/exercise are performed in a variety of ways (due to seasonal conditions, organizational effort, suitability for everyday use, etc.) and intensities, thus, the questions on the frequency, duration, skills level, etc., were adapted to each sport/exercise practice, and categorised afterwards. 

### 2.2. Procedures

Sport science students who were well connected to the respective sport scene, promoted the link to the survey within Austria. Sports associations and clubs were approached as well as athletes at training sites, camps and tournaments. Furthermore, social networks, such as Facebook, newsletters, blogs, as well as personal contacts to coaches and teams were used to increase the survey participation rate. In this way, sport/exercise maintainers who were strongly affiliated or connected to their respective sport/exercise were targeted. All data were collected anonymously through the online platform *LimeSurvey* and saved directly on the internal university server. On the first page of the survey, the potential participant was informed of the purpose and procedures of the study and asked for participation. All participants gave voluntary informed consent.

The overall survey sample included 12,301 participants. Participants under the age of 18 or over 65 (n = 1003) and participants that were practising the respective sport or exercise less than 1 h per week (n = 473) or for less than one year (n = 121), were excluded from further data analysis as the study targeted adult sport and exercise maintainers. 

The data were screened for unusual cases, anomalies and outliers and checked for any mistakes or extreme response patterns (e.g., highest agreement on all items). Multivariate outliers were identified through Mahalanobis distance and checked again for their response patterns. As a consequence, 58 participants were excluded from further data analysis. Thus, the final sample comprised 10,646 adult sport and exercise maintainers.

### 2.3. Statistical Analysis

Given the clearly defined model structure of the instrument [30,38], a confirmatory approach to data analysis was used. The data were not normally distributed, due to the use of the five-point Likert scale [38]. However, using the bootstrapping method (Bollen–Stine) the maximum likelihood estimation was considered as adequate [39,40]. By analogy with Lehnert et al. [38], covariance between the latent factors was permitted. For indicator-reliability the squared multiple correlations (SMC) of the items were used; acceptable values are >0.4. For factor reliability the AVE (average variance explained) >0.5 and CR (composite reliability) >0.5 were used [40,41]. The *χ*^2^ was expected to be significant and not an adequate indicator due to the large sample size [40,41]. Therefore, the model-fit-indices RMSEA (root mean square error of approximation; good values < 0.06), SRMR (standardised root mean square residual; good values < 0.08), CFI (Comparative Fit Index; good values > 0.95) and GFI (Goodness of Fit Index; good values > 0.95) were used. Comparing different models in the multi-group analysis, a change of 0.01 in the model-fit-indices RMSEA and CFI was considered meaningful [40,41]. In addition, modification indices of the corresponding measurement model were analysed to identify possible misfits. Univariate and descriptive analysis were performed with IBM SPSS Statistics for Windows v22.0 (IBM Corp., Armonk, NY, USA), and confirmatory factor analysis and multi-group analysis with IBM SPSS Amos v24.0 (IBM Corp., Armonk, NY, USA). 

Firstly, the adaptation of the original BMZI for adult sport practitioners (suggested by [30]) by the exclusion of the two items *comper4* (“for the thrill”) and *actenj2* (“primarily out of joy of movement”) was tested with the larger sample of this study. These two items showed low indicator-reliability (0.23 and 0.22) and high covariance with several items of other factors. Furthermore, the *Contact* factor was split in two: *Contact in sport* and *Contact through sport*. This was done, as recommended by Ley and Krenn [30], to possibly distinguish between *Contact in sport* and *Contact through sport* motives; cf. [21,38]. Thus, in this study the modified measurement model of the BMZI with 22 items and an eight-factorial structure was tested using confirmatory factor analysis. 

Secondly, multi-group analysis was performed to test the model for invariance regarding gender, age and years of sport/exercise experiences. Therefore, three age groups were established according to emerging adulthood (18–25 years), early adulthood (26–40 years) and middle adulthood (41–65 years); cf. [21], and three groups regarding years of sport/exercise experiences: short- (1–3 years), medium- (4–10 years) and long-term (>10 years) maintainers. Increasingly restrictive multi-group models were tested. In the metric model, the factor loadings were constrained to be equal, and in the scalar model, in addition, the intercepts of the indicators were set equal across the groups. 

Thirdly, motive value means were analysed to describe the sport and exercise maintainers’ motives and to test for correlations and differences according to various variables, i.e., gender, age, years of practise, and sport and exercise types. For the purpose of exploring differences among the sport and exercise types, the 40 sports and exercise practices were classified in team sports, individual sports with a direct opponent and individual sports/exercises with no direct opponent (see Sample description). The Mann–Whitney U-test and the Kruskal–Wallis test were used to test for significant differences and the Pearson correlation coefficient was used for linear correlation of the motive values with age and with years of sport/exercise experiences. 

## 3. Results

### 3.1. Participants Characteristics

The mean age of the 10,646 participants was 32.4 (±11.1) years. The sample included maintainers from a broad range of sports and exercise practices (n = 40). The respective sport and exercise was done for 12.6 (±11.1) years on average. A total of 47% reported that they did not participate in competitions; 8% assessed themselves at being at a beginner stage, 32% at an intermediate, 46% at an advanced and 14% at a professional/master stage. Regarding the highest educational achievement, 44.4% stated an academic degree and 36.3% stated a high school certificate. Table 1 displays characteristics of study participants according to gender.

### 3.2. Model Fit and Measurement Invariance

Confirmatory factor analysis of the proposed model yielded a good model fit (*χ*^2^(181) = 4909.88, *p* ˂ 0.001, *χ^2^/df =* 27.13; *p_Bollen-Stine-Bootstrap_* = 0.002, RMSEA = 0.050 (0.048–0.051), SRMR = 0.043, CFI = 0.960, GFI = 0.958). Table 2 displays the descriptive statistics, factor loadings of the items and factor reliability and correlations. The indicator reliability and standardised factor loadings were satisfactory (>0.40), except item *comper3* “to achieve sport/exercise goals” (SMC = 0.32). The modification indices displayed that the error variable of this item also showed high covariance with *fithea1* (“to keep myself in good physical shape”) and *fithea2* (“primarily to be fit”) as well as moderate covariance with *figapp3* (“because of my body shape”).

Multi-group analyses were conducted to test configural, metric and scalar invariance for gender, age and years of sport/exercise experiences. 

The female and the male models had identical measurement concepts and acceptable model fits (see Table 3). The *unconstrained* models showed satisfactory values. Furthermore, both models showed similar to identical indicator reliabilities. The metric model and the scalar model indicate towards measurement invariance. Looking for possible misfits, the modification indices of the measurement intercepts model (scalar model) indicated that item *comper2* (“to compete with others”) had problematic values.

All three age models showed identical measurement concepts and acceptable model fits (see Table 4). The *unconstrained* model had acceptable values. Furthermore, all models showed similar to identical indicator reliabilities. Metric and scalar invariance can be assumed. 

Furthermore, metric and scalar model invariance regarding years of sport/exercise experiences can be assumed. All models had identical measurement concepts and acceptable model fits and the *unconstrained* model showed acceptable values (see Table 5). Similar to identical indicator reliabilities were observed across the models. Yet, analysing the modification indices, the values of items *comper3* (“to achieve sport/exercise goals”) and *figapp3* (“because of my body shape”) indicated towards slight misfits in the intercepts model. 

### 3.3. Participation Motives, Group Differences and Correlations

For describing the participation motives across different groups, the means of the motive values were compared according to the variables: gender, age, years of sport/exercise experiences and sport/exercise types. Significant differences were found regarding gender (see Table 6). The male participants (*n* = 6088) valued *Competition/Performance* as more important than female participants (*n* = 4558). Nevertheless, *Activation*, *Fitness/Health* and *Aesthetics* were the highest scored motives among the female and the male participants. Furthermore, both gender groups gave more importance to *Contact in sport* than to *Contact through sport*.

The measurement detected correlations between the motives and age. *Contact in sport* (*r* = −0.11, *p* < 0.001), *Contact through sport* (*r* = −0.10, *p* < 0.001), *Competition/Performance* (*r* = −0.21, *p* < 0.001) and *Distraction/Catharsis* (*r* = −0.11, *p* < 0.001) negatively correlated with age; meanwhile *Fitness/Health* (*r* = 0.13, *p* < 0.001)*, Body/Appearance* (*r* = 0.07, *p* < 0.001), *Aesthetics* (*r* = 0.05, *p* < 0.001) and *Activation* (*r* = 0.07, *p* < 0.001) correlated positively with age, although the correlation coefficient values were rather low. 

The measurement also detected correlations between the motives and years of sport/exercise experiences. *Contact in sport* (*r* = 0.16, *p* < 0.001), *Aesthetics* (*r* = 0.06, *p* < 0.001) and *Activation* (*r* = 0.04, *p* < 0.001) were positively related, meanwhile *Competition/Performance* (*r* = −0.03, *p* < 0.001), *Body/Appearance* (*r* = −0.18, *p* < 0.001), *Fitness/Health* (*r* = −0.19, *p* < 0.001) and *Distraction/Catharsis* (*r* = −0.10, *p* < 0.001) were negatively related with years of sport/exercise experiences. No significant correlation was found with *Contact through sport* (*r* = 0.01, *p* = 0.60). The correlation coefficient values were low, particularly in *Aesthetics, Activation* and *Competition/Performance*. In the latter no differences among the three groups (short-term, medium-term and long-term maintainers) were found (H = 0.113; *p* = 0.945). 

In addition, the analysis detected differences among sport/exercise types (see Table 7). Those who played team sports (n = 2318) valued the *Contact* factors and *Competition/Performance* more highly; those who played individual sports with a direct opponent (n = 1453) valued *Body/Appearance* and *Fitness/Health* more highly; and those who did individual sports/exercise with no direct opponent (n = 6875) valued *Activation* and *Aesthetics* more highly.

## 4. Discussion

The purpose of the study was to contribute to the provision of a validated questionnaire that allows measuring motive differences among age, gender and years of sport/exercise, and to the understanding of the goal contents of (diverse groups of) sport and exercise maintainers and, thus, of the subjective incentives of the respective sport and exercise practice. A slightly modified version of the BMZI for adult sport practitioners [30] was examined with data from 10,646 sport and exercise maintainers from 40 different sports and exercise practices. The confirmatory factor analysis confirmed the hypothesis of an acceptable model fit and measurement invariance. The good model fit aligns with similar findings of acceptable to good model fits of the BMZI and modified versions [23,38]. Furthermore, the multi-group analysis showed metric and scalar measurement invariance with regard to gender, age and years of sport/exercise experiences. The scalar measurement invariance indicates that the modified BMZI can be used to conduct meaningful comparisons across these groups. 

Nevertheless, modification indices were analysed to identify possible misfits. The *Competition/Performance* factor items seemed partially problematic. This may be caused by this factor including both, more intrinsic elements of challenge and self-orientation and more extrinsic elements of social recognition through competition. Deleting the problematic item *comper3* in the confirmatory factor analysis would improve model fit indices. On the whole, however, the factor would no longer represent the diverse aspects of competition and performance. Thus, when aiming to capture a more complete range of motives, both dimensions, i.e., competition/performance with social reference (e.g., social recognition) and competition/performance in relation to oneself (e.g., to steadily improve one’s own performance) could be included as two different factors; cf. Gabler's motive taxonomy [21]. 

The measurement model differentiated *Contact* into two dimensions, which proved to be useful in depicting mean differences. The results show that *Contact in sport* was valued as more important than *Contact through sport* in sport maintainers. Furthermore, *Contact in sport* was positively related with years of sport/exercise experiences; cf. [8]. This finding may not be surprising, as one may assume that more novice sport performers rather expect to do new friends as they start to participate in the sport; meanwhile more long-term sport maintainers may rather aim to ‘maintain’ their friendships; cf. [32]. Furthermore, *Contact in sport* (‘To be social with others’, ‘To do something in a group’, ‘To meet friends/acquaintances’) refers to experiencing social interaction in sport, which contains social affiliation and identity building processes and, thus, is rather self-rewarding, pointing towards more intrinsic goal contents. *Contact through sport* (‘To get to know people’, ‘To make new friends through exercise’) refers more to making new contacts and thus include a rather outward orientation [18,24,35]. 

The factors *Activation*, *Fitness/Health* and *Aesthetics* as well as *Contact in Sport* were the most highly valued motives in sport and exercise maintainers. These factors contain more intrinsic goal contents [18,24,35]. These results are in line with studies that showed that fitness and health motives predict intrinsic motivation [8,10] and are valued higher among active people compared to inactive people [7]. *Body/Appearance* and *Contact through sport*, which contain more extrinsic contents, were less valued; cf. appearance/image in [35]. In total, the findings confirm the hypothesis that sport and exercise maintainers pursue predominantly goal contents related to intrinsic motivations.

Furthermore, the analysis detected mean differences across gender, age and years of sport/exercise experiences as well as across sports/exercise types as hypothesised. These findings align with other studies [25,26,27,30,38]. Female sport maintainers gave more importance to motives that relate to *Activation*, *Distraction/Catharsis*, *Health/Fitness* and *Body/Appearance* than men. This finding relates to other studies showing women to give more importance to psychological benefits, physical condition and physical appearance [6,26,27]. Notably, the male and the female group valued the subjective importance of some factors (e.g., *Competition/Performance*) differently; but both groups gave the highest scores to the factors *Activation*, *Fitness/Health* and *Aesthetics*. Furthermore, those who did the sport/exercise for a shorter time valued the outcome-related motives, such as *Fitness/Health* and *Body/Appearance*, more highly; indicating that more novice sport performers may be more attracted to sport by values related to healthism; cf. [32]. In contrast, *Contact in sport, Aesthetics* and *Activation*, which are rather task and action-related and intrinsic motives, correlated slightly positive with years of sport/exercise experiences. This is supported by other studies indicating that intrinsic motivation is crucial for sport maintenance [7,8]. *Aesthetics*, *Activation* and *Distraction/Catharsis* were ranked higher by those who did individual exercise/sports. *Contact* and *Competition/Performance* seem central in team sports and for younger people. Importance of *Fitness/Health* increased with age. This may be explained by the increasing health problems and concerns of elder people [6,14,31]. These results support previous research, highlighting similarities as well as differences according to gender, age and sport type.

This study comes with limitations. The grouping of sports/exercise was based on objective categories (e.g., individual/team sports). However, further analysis of the data may yield clusters of sports according to motive profiles and thus, classify sports/exercises according to the subjective goal contents of their practitioners. The confirmatory analysis was limited to the BMZI items and factors. Sport-specific motive items that were added in each sport-specific questionnaire were not included. It is probable that other important motives were missed out. Future research could explore further items and factors to capture separately the intrinsic and extrinsic goal contents of competition/performance. An additional factor capturing motives relating to lived experiences during sport and exercise practices (mastery, task and action-related goal contents) could be added separately from the *Activation* factor; cf. [23,30]. Depending on the user’s purpose, the inventory can be used to cover the broad range of participation motives [36,38] or aligned to SDT by reducing the items to those more strictly adhering to goal contents and not to regulatory processes of goal pursuits [23,37]. Furthermore, the sample was not stratified and results may be cofounded by other characteristics not analysed in this study.

It has been pointed out that the motives of inactive and active people differ [7]; however, Geller et al. [8] also found out that there were no significant differences between maintainers (with over 10 years regular physical activity) and improvers (improving physical activity levels over the last 10 years), “which may indicate similar motives when both adopting and maintaining physical activity” (p. 65). The current study showed that years of sport/exercise experiences correlated to some extend positively with *Contact in sport* and negatively with *Fitness/Health* and *Body/Appearance*. If we assume that maintainers’ goal contents relate to their experiences; cf. [9], it can be expected that people, who are getting active, probably will make similar experiences in the recommended sport and exercise as maintainers, re-evaluate expectations and change the importance of the diverse participation motives over the time with gaining experiences. Thus, recommending sport and exercises based on the goal contents of sport/exercise maintainers probably contribute to a maintained participation and adherence to sport and exercise. However, longitudinal research is needed to confirm the actual changes in participation motives over time with gaining experiences and the impact on adherence. 

## 5. Conclusions

This study contributes to the validation of the *Bernese Motive and Goal Inventory* and to the knowledge of the participation goal contents of maintainers. The tested inventory can be applied in sport and exercise maintainers independently of gender, age and years of sport/exercise experiences. It can be used to identify participation motives of individuals or population groups, or to describe the sports/exercise by the goal contents of their maintainers. This is important, as the study highlights similarities as well as differences according to gender, age and sport type. Thus, individual or group-specific tailoring of sport and exercise programmes and recommendations for the promotion of maintained participation is required. Gender and age specific motives must be considered as well as different incentives of the diverse types of sport/exercises. This information may be used by sport managers and trainers to design *fitting* interventions, services and products, i.e., that satisfy to a higher degree the needs and motives of their participants. Sport service providers may make use of the inventory to assess the motives of their participants and then to provide individualised or group-tailored interventions or recommendations. Likewise, fitness apps may automatically adapt information provision, prompts and feedback according to user’s participation motive profile, considering differences among gender and age groups. This may contribute to reduce dropout rates and fluctuation in sport/exercise practices.

## Figures and Tables

**Table 1:** Characteristics of study participants according to gender (women, men).

	Women	Men
Overall (%, n)	42.8% (n = 4558)	57.2% (n = 6088)
Age (years; mean (95%CI))	31.0 (30.7–31.3)	33.5 (33.2–33.7)
Emerging adulthood (18–25 years) (%, n)	50.5% (1762)	49.5% (1725)
Early adulthood (26–40 years) (%, n)	40.7% (1938)	59.3% (2827)
Middle adulthood (41–65 years) (%, n)	35.8% (858)	64.2% (1536)
Years of practice (years; mean (95%CI))	11.2 (10.9–11.5)	13.6 (13.3–13.8)
Short-term maintainers (1–3 years) (%, n)	51.1% (1308)	48.9% (1250)
Medium-term maintainers (4–10 years) (%, n)	43.5% (1425)	56.5% (1853)
Long-term maintainers (41–65 years)	37.9% (1825)	62.1% (2985)
Type of sports/exercise practice		
Team sports (%, n)	28.8% (668)	72.2% (1650)
Individual sport with opponent (%, n)	35.0% (508)	65.0% (945)
Individual sport without opponent (%, n)	49.2% (3382)	50.8% (3493)

**Table 2:** Descriptive statistics, factor loadings, factor reliability and correlations.

	Descriptive		Reliability	Standardised Factor Loadings							
**Item**	**M**	**SD**	**SMC**	**Ci**	**Ct**	**C/P**	**B/A**	**F/H**	**A**	**D/C**	**A**
con1	3.28	1.32	0.77	0.88							
con2	3.01	1.33	0.67	0.82							
con3	3.29	1.31	0.69		0.83						
con4	2.65	1.24	0.78		0.88						
con5	2.59	1.24	0.79		0.89						
comp1	2.27	1.38	0.70			0.84					
comp2	2.43	1.34	0.70			0.84					
comp3	3.28	1.34	0.32			0.56					
figapp1	1.93	1.21	0.74				0.86				
figapp2	2.20	1.31	0.86				0.93				
figapp3	2.33	1.33	0.67				0.82				
fithea1	3.79	1.17	0.66					0.82			
fithea2	3.57	1.24	0.75					0.87			
fithea3	2.82	1.28	0.47					0.68			
aes1	3.09	1.38	0.44						0.66		
aes2	3.50	1.29	0.62						0.79		
discat1	2.88	1.35	0.56							0.74	
discat2	2.85	1.34	0.44							0.66	
discat3	3.56	1.23	0.66							0.81	
discat4	3.14	1.32	0.54							0.73	
actenj1	3.54	1.23	0.51								0.71
actenj3	3.85	1.11	0.52								0.72
	Descriptive		Reliability		Inter-correlations						
Factor	M	SD	CR	AVE	Ct	C/P	B/A	F/H	A	D/C	A
Ci	3.19	1.18	0.88	0.71	0.73	0.30	−0.08	0.01	0.26	0.09	0.16
Ct	2.62	1.17	0.88	0.79		0.38	0.05	0.12	0.26	0.22	0.19
C/P	2.66	1.13	0.80	0.57			0.12	0.14	0.05	0.11	−0.12
B/A	2.15	1.17	0.90	0.76				0.60	0.01	0.27	0.09
F/H	3.40	1.06	0.83	0.63					0.25	0.35	0.31
A	3.29	1.17	0.69	0.53						0.28	0.50
D/C	3.11	1.06	0.83	0.55							0.77
A	3.70	1.02	0.68	0.51							

Ci = Contact in sport; Ct = Contact through sport; C/P = Competition/Performance; B/A = Body/Appearance; F/H = Fitness/Health; A = Aesthetics; D/C = Distraction/Catharsis; A = Activation; SMC = squared multiple correlations; CR = composite reliability; AVE = average variance explained.

**Table 3:** Analysis of invariance across gender groups.

Model	*χ* ^2^	*df*	RMSEA	SRMR	CFI	Diff. *RMSEA*	Diff. *CFI*
Female (n = 4558)	2563.30	181	0.054 (0.052–0.056)	0.048	0.954		
Male (n = 6088)	2670.26	181	0.048 (0.046–0.049)	0.039	0.961		
Configural model	5233.58	362	0.036 (0.035–0.036)	0.048	0.958	-	-
Metric model	5323.98	376	0.035 (0.034–0.036)	0.048	0.957	−0.001	−0.001
Scalar model	6669.77	398	0.038 (0.038–0.039)	0.051	0.946	+0.002	−0.012

**Table 4:** Analysis of invariance across age groups: emerging, early and middle adulthood.

Model	*χ* ^2^	*df*	RMSEA	SRMR	CFI	Diff. *RMSEA*	Diff. *CFI*
Emerging (n = 3487)	1793.92	181	0.051 (0.048–0.053)	0.043	0.957		
Early (n = 4765)	2439.08	181	0.051 (0.049–0.053)	0.046	0.957		
Middle (n = 2394)	1073.24	181	0.045 (0.043–0.048)	0.044	0.966		
Configural model	7469.17	637	0.032 (0.031–0.032)	0.047	0.941	-	-
Metric model	7491.89	651	0.031 (0.031–0.032)	0.047	0.941	−0.001	0.000
Scalar model	7622.13	673	0.031 (0.031–0.032)	0.047	0.940	−0.001	−0.001

**Table 5:** Analysis of invariance regarding years of sport/exercise experiences.

Model	*χ* ^2^	*df*	RMSEA	SRMR	CFI	Diff. *RMSEA*	Diff. *CFI*
Short-term (1–3 y)	1623.13	181	0.056 (0.054–0.059)	0.053	0.947		
Medium-term (4–10 y)	1527.48	181	0.048 (0.045–0.050)	0.041	0.964		
Long-term (>10 y)	2009.72	181	0.046 (0.044–0.048)	0.037	0.964		
Configural model	5160.40	543	0.028 (0.028–0.029)	0.053	0.960	-	-
Metric model	5282.93	571	0.028 (0.027–0.029)	0.052	0.959	0.000	−0.001
Scalar model	7208.09	615	0.032 (0.031–0.032)	0.057	0.943	+0.004	−0.017

**Table 6:** Comparison of motive mean values across gender.

		M	*SD*	U	*p*	*d*
*Contact in sport*	Male	3.25	1.13	14,634,235.5	<0.001	0.094
	Female	3.12	1.25			
*Contact through sport*	Male	2.71	1.14	15,489,409.5	<0.001	0.20
	Female	2.49	1.20			
*Competition/Performance*	Male	2.84	1.14	16,854,173	<0.001	0.374
	Female	2.42	1.07			
*Aesthetics*	Male	3.29	1.14	13,824,121	0.746	-
	Female	3.29	1.21			
*Body/Appearance*	Male	2.04	1.09	12,337,947	<0.001	0.191
	Female	2.31	1.25			
*Fitness/Health*	Male	3.33	1.06	12,743,418	<0.001	0.14
	Female	3.48	1.05			
*Distraction/Catharsis*	Male	3.01	1.05	12,179,126	<0.001	0.211
	Female	3.24	1.06			
*Activation*	Male	3.59	1.02	11,782,368	<0.001	0.261
	Female	3.84	1.00			

**Table 7:** Comparison of motive mean values across sport/exercise types.

		M	*SD*	H	*p*	*d*
*Contact in sport*	Team	3.90	0.89	1132.4	<0.001	0.689
	Opponent	3.12	1.12			
	Individual	2.97	1.19			
*Contact through sport*	Team	3.13	1.08	652.9	<0.001	0.51
	Opponent	2.70	1.16			
	Individual	2.43	1.15			
*Competition/Performance*	Team	3.48	1.00	1824.0	<0.001	0.909
	Opponent	2.90	1.14			
	Individual	2.33	1.01			
*Aesthetics*	Team	3.07	1.12	129.9	<0.001	0.221
	Opponent	3.28	1.19			
	Individual	3.37	1.17			
*Body/Appearance*	Team	2.07	1.03	52.4	<0.001	0.138
	Opponent	2.33	1.16			
	Individual	2.15	1.21			
*Fitness/Health*	Team	3.28	0.95	194.0	<0.001	0.271
	Opponent	3.74	0.92			
	Individual	3.36	1.11			
*Distraction/Catharsis*	Team	3.00	1.03	39.5	<0.001	0.119
	Opponent	3.10	1.09			
	Individual	3.15	1.06			
*Activation*	Team	3.36	0.97	526.2	<0.001	0.455
	Opponent	3.51	1.06			
	Individual	3.85	0.99			
